# Patient Experience Shows Little Relationship with Hospital Quality Management Strategies

**DOI:** 10.1371/journal.pone.0131805

**Published:** 2015-07-07

**Authors:** Oliver Groene, Onyebuchi A. Arah, Niek S. Klazinga, Cordula Wagner, Paul D. Bartels, Solvejg Kristensen, Florence Saillour, Andrew Thompson, Caroline A. Thompson, Holger Pfaff, Maral DerSarkissian, Rosa Sunol

**Affiliations:** 1 Department of Health Services Research and Policy, London School of Hygiene and Tropical Medicine, London, United Kingdom; 2 Department of Epidemiology, The Fielding School of Public Health, University of California, Los Angeles (UCLA), Los Angeles, California, United States of America; 3 Academic Medical Center, University of Amsterdam, Amsterdam, the Netherlands; 4 Netherlands Institute for Health Services Research, Utrecht, the Netherlands; 5 Department of Public and Occupational Health, VU University Medical Center, Amsterdam, the Netherlands; 6 Danish Clinical Registries, Aarhus, Denmark, and Department of Clinical Medicine, Aalborg University, Aalborg, Denmark; 7 Unité Méthodes Evaluation en Santé, Centre Hospitalier Universitaire de Bordeaux, Bordeaux, France; 8 School of Social and Political Science, University of Edinburgh, Edinburgh, United Kingdom; 9 Palo Alto Medical Foundation Research Institute (PAMFRI), Palo Alto, California, United States of America; 10 Institute of Medical Sociology, Health Services Research and Rehabilitation Science, University of Cologne, Cologne, Germany; 11 Avedis Donabedian Research Institute (FAD), Universitat Autonoma de Barcelona, Barcelona, Spain; 12 Red de investigación en servicios de salud en enfermedades crónicas REDISSEC, Barcelona, Spain; University of Geneva, SWITZERLAND

## Abstract

**Objectives:**

Patient-reported experience measures are increasingly being used to routinely monitor the quality of care. With the increasing attention on such measures, hospital managers seek ways to systematically improve patient experience across hospital departments, in particular where outcomes are used for public reporting or reimbursement. However, it is currently unclear whether hospitals with more mature quality management systems or stronger focus on patient involvement and patient-centered care strategies perform better on patient-reported experience. We assessed the effect of such strategies on a range of patient-reported experience measures.

**Materials and Methods:**

We employed a cross-sectional, multi-level study design randomly recruiting hospitals from the Czech Republic, France, Germany, Poland, Portugal, Spain, and Turkey between May 2011 and January 2012. Each hospital contributed patient level data for four conditions/pathways: acute myocardial infarction, stroke, hip fracture and deliveries. The outcome variables in this study were a set of patient-reported experience measures including a generic 6-item measure of patient experience (NORPEQ), a 3-item measure of patient-perceived discharge preparation (Health Care Transition Measure) and two single item measures of perceived involvement in care and hospital recommendation. Predictor variables included three hospital management strategies: maturity of the hospital quality management system, patient involvement in quality management functions and patient-centered care strategies. We used directed acyclic graphs to detail and guide the modeling of the complex relationships between predictor variables and outcome variables, and fitted multivariable linear mixed models with random intercept by hospital, and adjusted for fixed effects at the country level, hospital level and patient level.

**Results:**

Overall, 74 hospitals and 276 hospital departments contributed data on 6,536 patients to this study (acute myocardial infarction n = 1,379, hip fracture n = 1,503, deliveries n = 2,088, stroke n = 1,566). Patients admitted for hip fracture and stroke had the lowest scores across the four patient-reported experience measures throughout. Patients admitted after acute myocardial infarction reported highest scores on patient experience and hospital recommendation; women after delivery reported highest scores for patient involvement and health care transition. We found no substantial associations between hospital-wide quality management strategies, patient involvement in quality management, or patient-centered care strategies with any of the patient-reported experience measures.

**Conclusion:**

This is the largest study so far to assess the complex relationship between quality management strategies and patient experience with care. Our findings suggest absence of and wide variations in the institutionalization of strategies to engage patients in quality management, or implement strategies to improve patient-centeredness of care. Seemingly counterintuitive inverse associations could be capturing a scenario where hospitals with poorer quality management were beginning to improve their patient experience. The former suggests that patient-centered care is not yet sufficiently integrated in quality management, while the latter warrants a nuanced assessment of the motivation and impact of involving patients in the design and assessment of services.

## Introduction

Patient-centered care is increasingly considered as an integral component of quality of care [1–3]. It is often defined as “health care that establishes a partnership among practitioners, patients, and their families (when appropriate) to ensure that decisions respect patients’ wants, needs, and preferences and that patients have the education and support they need to make decisions and participate in their own care” [4]. Patient-centered care denotes a complex construct and embraces many different principles and activities, such as affording patients dignity, compassion and respect, offering coordinated care, support or treatment, offering personalized care, support or treatment and supporting patients to recognize and develop their own strengths and abilities to enable them to live an independent and fulfilling life [5–7]. While an important goal in itself, patient-centered care is also a means to an end as it is consistently related with other desirable outcomes, such as clinical effectiveness and patient safety [8, 9]. The focus on patient centered care is not entirely new and numerous contributions to the literature have stressed the need to improve patient-centered care to ensure dignity, trust, involvement in decision-making, and improved outcomes [10–13]. Yet, patient-centeredness has traditionally received less attention than other dimensions of health care quality.

The level of patient-centeredness is usually assessed using patient-reported experience measures (PREMs) and these measures are increasingly being used to routinely monitor the quality of care. For example, efforts are underway in England’s National Health Service (NHS) to introduce a national reporting system for PREMS, similar to the Patient-reported Outcomes (PROMS) initiative that already collects and publicly reports on patient level data for four elective surgical procedures [14, 15]. In the United States (US), standardized, national data using the Hospital Consumer Assessment of Healthcare Providers and Systems (HCAHPS) survey has been collected and publicly reported over several years [16]. More recently, reimbursement as part of the Value-Based Purchasing Scheme links a portion of a hospital’s payment from the Centers for Medicare and Medicaid Services (CMS) to a set of quality measures, including the HCAPHS score [17, 18], reinforcing the focus on patient-centered care.

Although a patient-centered approach is widely advocated, hospital performance on PREMs varies substantially. There is evidence that patients frequently do not receive important information on their condition and options for self-management, and that there is insufficient involvement of patients in developing quality goals [19, 20]. Moreover, surveys frequently report patients’ dissatisfaction with the way services are organized in the hospital, the lack of time for consultations, and difficulties in understanding what doctors tell them [21, 22]. This has implications beyond improving the humanity of care and affects other quality of care outcomes, such as adherence to medication, increased utilization of health services, occurrence of infections, or unnecessary readmissions after a hospitalization [9].

With the increasing attention given to PREMs, hospital management needs to understand ways of improving patient-centeredness of their organizations. It is currently unclear whether hospitals with more mature quality management systems or stronger focus on patient involvement and patient-centered care strategies perform better on PREMs [23, 24]. The objective of this research is to assess these complex relationships.

## Methods

### Study design, setting and population

This study was conducted as part of the “Deepening our understanding of quality improvement in Europe (DUQuE)” project, which was funded by the European Union’s (EU) 7th Research Framework Programme [25]. The overall aim of the project was to study the relationship between organizational quality improvement systems and organizational culture, professional involvement and patient empowerment at hospital and departmental level, and the impact of these constructs on the quality of care delivered, measured in terms of clinical effectiveness, patient safety and patient experience.

The objectives of the overall project and the different constructs that were assessed within the DUQuE project are described in detail elsewhere [26]. In brief, the DUQuE study is based on a conceptual model addressing four levels: hospital, departmental (or pathway) level, patient's level and external factors influencing uptake of management decisions). Hospital and departmental wide factors assessed include quality management systems, organizational culture, professional involvement, external pressure and patient involvement. Patient level processes and outcomes were assessed for four selected conditions (acute myocardial infarction (AMI), obstetrical deliveries, hip fracture and stroke). These conditions were chosen because they cover an important range of types of care, there are evidence-based standards for process of care against which compliance could be assessed and there is demonstrated variability in both compliance with process of care measures and outcomes of care (complications, mortality) that would allow for analysis of associations between these measured constructs.

We employed a cross-sectional, multi-level study design in which patient-level measurements are nested in hospital departments, which are in turn nested in hospitals in 7 EU countries. Selected countries had to have a sufficient number of hospitals to fulfil sampling criteria, represent varied approaches to financing and organizing health care, have research staff with experience in conducting comprehensive field tests and represent the geographical reach of the EU. Turkey was included because of the status of its EU candidacy at the start of the project. The countries invited to participate in the field test were the Czech Republic, England, France, Germany, Poland, Portugal, Spain and Turkey.

### Outcomes, predictors and covariates

The outcome variables in this study are a set of PREMs collected at patient level. We developed a questionnaire that included a generic 6-item measure of patient experience (The Nordic Patient Experience Questionnaire) [27] and a 3-item measure of patient-perceived discharge preparation (Health Care Transition Measure) [28], supported by two single item measures on perceived involvement in care [19] and hospital recommendation [16]. All four measures were assessed for each group of patients (AMI, Stroke, hip fracture, deliveries).

Predictor variables include three measures: First, a measure of the maturity of the hospital quality management system assessed by a questionnaire administered to the hospital quality manager. Second, a measure of departmental strategies for the involvement of patients or their representatives in quality management functions assessed by a questionnaire administered to the head of the department. Third, a measure of the implementation of patient-centered care strategies, assessed by an external visit to the department. These measures build on previously validated tools (Table 1), [29–31].

**Table 1:** Constructs, measure domains and data collection methods used in this study.

Assessment level	Measure domain	Measure domain definition	Data collection method	Administration system
Hospital level	Quality Management System Index (QMSI)	Quality management system index (QMSI): a multi-dimensional index (9 dimensions, 46 items) on the implementation of quality management activities, covering quality policies, procedures and activities (such as quality monitoring, infection control, complaints handling etc.).	Questionnaire to hospital quality manager (QM)	Electronically administered questionnaire
	Patient involvement in quality management	A five-item index reflecting the extent to which patients or their representatives are involved in the development and design of processes, quality committees, quality improvement projects and discussion of results of quality improvement projects	Questionnaire to hospital quality manager	Electronically administered questionnaire
Pathway/department level	Patient involvement in quality management	A five-item index reflecting the extent to which patients or their representatives are involved in the development and design of processes, quality committees, quality improvement projects and discussion of results of quality improvement projects	Questionnaire to manager of care pathways or head of department	Electronically administered questionnaire
	Patient centered care strategies	A four-item score on the implementation of key strategies to improve patient centered care, incorporating existence of formal patient surveys, written policies on patients' rights, providing access to patient information literature and fact sheets for post-discharge care	Assessment at pathway or department settings performed by an external visitor	Both paper and electronically administered audit forms
Patient experience	Generic patient experience	Nordic Patient Experiences Questionnaire (NORPEQ): a generic 6-item measure on patient experience of the quality of hospital care, including confidence in doctors’ and nurses’ skills, patient-centeredness and information provision.	Patient survey	Paper-based questionnaire
	Perceived patient involvement	Perceived patient involvement: a single item measure on patients’ perceived involvement in care (from Commonwealth Fund sicker patients survey)	Patient survey	Paper-based questionnaire
	Hospital recommendation	Measure of hospital recommendation: a single item measure on the extent to which the patient would recommend the hospital to their family or friends (from HCAHPS)	Patient survey	Paper-based questionnaire
	Perceived continuity of care	Health care transition measure (HCT): a 3-item measure of the patient perceived discharge process from the hospital to the community, including preferences, self-efficacy and understanding the medication regime.	Patient survey	Paper-based questionnaire

Covariates including in the statistical analysis include country, hospital teaching status (teaching vs non-teaching), hospital size (<200, 200–500, 501–100, or >1000 beds), hospital ownership (public vs not public) and (at patient level) patient age, gender, level of health literacy (single item Health Literacy Screener), and education level (no education, primary education, secondary education, further education beyond school, or university level education). Further details on outcome and predictor variables are presented in Table 1.

### Data collection

General acute care hospitals (public or private, teaching or non-teaching) with a minimum hospital size of 130 beds were considered for inclusion into the study. Hospitals were required to have a sufficient volume of care to ensure recruitment of 30 patients per condition over a 4-month period (a sample frame of a minimum of 90 patients). Hospitals were randomly selected in the participating countries between May 2011 and January 2012. Each hospital contributed patient level data from four conditions/pathways: acute myocardial infarction (AMI), stroke, hip fracture and deliveries.

Hospital recruitment was based on a simple random sample on the basis, including an oversampling factor to account for withdrawal of participants. The sampling distribution was compared with overall hospital characteristics in the participating countries and showed no difference in terms of number of beds, ownership and teaching status. Chief executive officers (CEOs) of a total of 548 hospitals were approached to participate in the study, of which 192 (35%) agreed. The main reasons of declining to participate were related to time constraints, organizational aspects and the complexity of the study. Data from 188 hospitals in 7 participating countries were included in the final analysis. After significant efforts, hospitals in England were not included partly due to delays in obtaining ethical approval and also extensive difficulty recruiting hospitals. Similarly, it proved difficult to recruit hospitals in Germany to the study and only 4 hospitals from this country were included in the analysis. Data was collected using a bespoke IT platform. The overall response rate for the different questionnaires was between 75 and 100% for the assessed measures. Detailed sample size calculations and information on response rates have been reported previously [25, 26].

### Hypotheses and analytical strategy

We hypothesized that higher PREM scores are achieved in hospitals (i) with more mature quality management systems, (ii) that involve patients in quality management functions and (iii) that implement key patient-centered care strategies.

**Fig 1:**
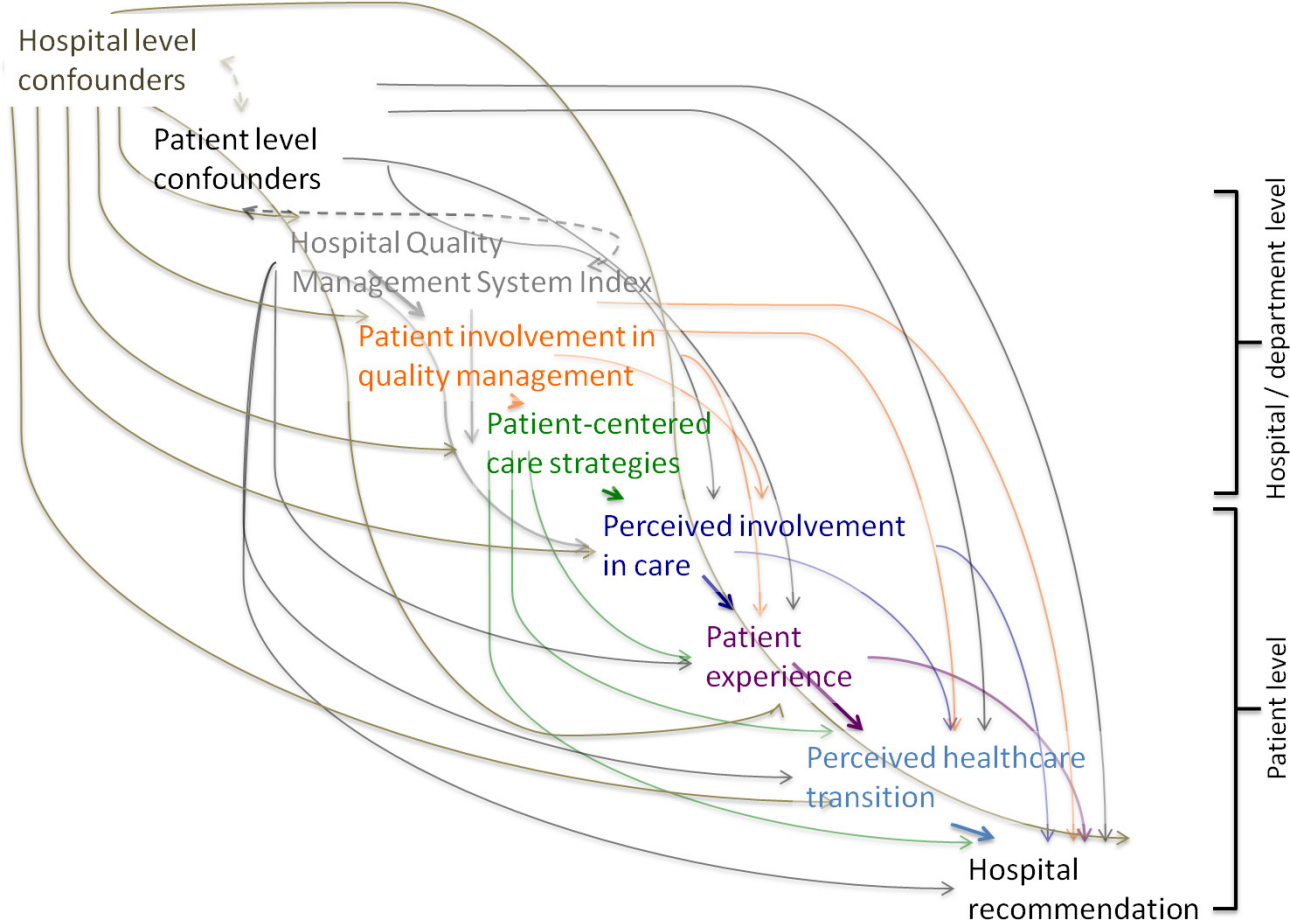
Directed acyclic graph of the relations between predictor and outcome variables. Note: A dashed bi-directed arrow represents the presence of an unmeasured common cause of the variables at the arrowhead. A variable at the tail of an arrow is considered a cause or a parent of the variable at the arrowhead. Alternatively, the arrow between any two variables can be read, in a non-causal way, as representing the flow of statistical information or the presence of statistical dependence between the two variables.

We used directed acyclic graphs (DAGs) to depict our knowledge and assumptions about the (plausible) interrelationships between the predictor and outcome variables. The edges in the DAG encode relations between predictors, outcomes and covariates and are governed by formal rules that can be used to guide the choice of covariates for confounding control [32, 33] (Fig 1).

According to our directed acyclic graph (Fig 1), in order (i) to estimate the effect of quality management systems on any PREM we adjusted for country as well as hospital-level and patient-level confounders; (ii) to estimate the effect of patient involvement in quality management on any PREM we adjusted for country, hospital-level and patient-level confounders, and quality management system index; and (iii) to estimate the effect of patient centered care strategies on any PREM we adjusted for country, hospital-level and patient-level confounders, quality management system index, and departmental-level patient involvement in QM. We estimated multivariable linear mixed models using PROC MIXED, with random intercept by hospital, and additionally adjusted for country, and fixed effects at the hospital level, and patient level in accordance with the DAG. All analyses were conducted using SAS version 9.3 (SAS Institute, Cary, NC, USA).

### Ethical and confidentiality

DUQuE fulfilled the requirements for research projects as described in the 7th framework of EU DG Research. Ethics approval was granted by the Department of Health of the Government of Catalonia, Spain. Data collection in each country complied with confidentiality requirements according to national legislation or standards of practice of that country. All data was anonymous and codes were used for hospitals and countries.

Minimum data supporting the data tables have been published in the public domain under http://dx.doi.org/10.6084/m9.figshare.1422011.

## Results

Overall, 276 departments from 74 hospitals contributed patient-level data to be included in this study. The majority were public hospitals (79.7%) and about half (44.5%) were teaching hospitals. Larger hospitals with more than 500 beds accounted for more than half of the hospitals in the sample (Table 2).

**Table 2:** Characteristics of Hospitals participating in study.

Characteristic	N	%
All Hospitals	74	
Czech Republic	12	(16.2)
France	11	(14.8)
Germany	4	(5.4)
Poland	12	(16.2)
Portugal	11	(14.8)
Spain	12	(16.2)
Turkey	12	(16.2)
Teaching Hospitals	33	(44.5)
Public Hospitals	59	(79.7)
Approximate number of beds in hospital		
<200	7	(9.4)
200–500	22	(29.7)
501–1000	31	(41.8)
>1000	14	(18.9)

6536 patients contributed sufficient data to the patient survey with an overall response rate of 75% [26]. The age and gender distribution is typical for the four conditions. Educational level is low for patients with hip fracture and stroke, reflecting the proportion of very old women in the study affected by these conditions. This is consistent with the values of the single-item health literacy screener, suggesting that hip fracture and stroke patients in the study have a low level of health literacy (Table 3).

**Table 3:** Characteristics of patient survey respondents.

Characteristics	Acute Myocardial Infarction		Deliveries		Hip Fracture		Stroke	
Total number of respondents, N (%)	1379	(21.0)	2088	(31.9)	1503	(22.9)	1566	(23.9)
Gender, N (%)								
Female	377	(27.3)	2057	(98.5)	1008	(67.0)	688	(43.9)
Male	952	(69.0)	NA	NA	427	(28.4)	830	(53.0)
Missing	50	(3.6)	31	(1.4)	68	(4.5)	48	(3.0)
Education level, N (%)								
No education	122	(8.8)	52	(2.4)	268	(17.8)	225	(14.3)
Primary education	431	(31.2)	218	(10.4)	656	(43.6)	590	(37.6)
Secondary education	507	(36.7)	859	(41.1)	364	(24.2)	476	(30.3)
Beyond school	177	(12.8)	449	(21.5)	102	(6.7)	136	(8.6)
University	102	(7.3)	469	(22.4)	61	(4.0)	104	(6.6)
Missing	40	(2.9)	41	(1.9)	52	(3.4)	35	(2.2)
Age (years), Mean (SD)	63.1	(12.9)	29.3	(5.8)	76.0	(13.2)	68.1	(13.1)
Current health state^1^								
Very good	208	(15.0)	764	(36.5)	126	(8.3)	147	(9.3)
Good	685	(49.6)	1088	(52.1)	632	(42.0)	680	(43.4)
Fair	401	(29.0)	207	(9.9)	599	(39.8)	609	(38.8)
Poor or very poor	57	(4.1)	11	(0.5)	122	(8.1)	117	(7.4)
Health literacy^1^	1.45	(1.7)	0.86	(1.4)	1.97	(1.7)	1.81	(1.7)

^1^On a scale from 0–4, 0 meaning “none at all” and 4 meaning “to a very large extent”, how much help do you need when you read instructions, pamphlets or other written material from your doctor or pharmacy?

In Table 4 we describe descriptive statistics for predictor and outcome variables.

**Table 4:** Descriptive statistics for predictor and outcome variables.

	AMI		Deliveries		Hip fracture		Stroke	
	Mean	(SD)	Mean	(SD)	Mean	(SD)	Mean	(SD)
Predictor Variables (Scale range)								
Quality Management Systems Index (Hospital Level) (0–27)	19.1	(3.8)	19.2	(4.1)	19.3	(4.0)	19.4	(4.1)
Patient Involvement in Quality Management (Pathway Level) (0–3)	0.8	(0.7)	0.6	(0.8)	0.6	(0.6)	0.6	(0.6)
Patient centered care strategies (Pathway Level) (0–4)	2.9	(0.8)	3.0	(0.8)	2.7	(0.8)	2.9	(0.8)
Outcome Variables (PREMs) (Scale range)								
Patient experience—NORPEQ (0–100)	86.8	(13.3)	85.4	(14.3)	79.3	(16.2)	83.2	(14.8)
Perceived patient involvement (0–4)	2.9	(1.1)	3.1	(0.9)	2.7	(1.1)	2.8	(1.1)
Perceived healthcare transitions—HCT (0–100)	79.8	(17.4)	81.8	(17.2)	74.6	(17.8)	77.8	(18.3)
Hospital recommendation (0–4)	3.6	(0.6)	3.4	(0.7)	3.3	(0.8)	3.4	(0.7)

The overall mean score of the Quality Management Systems Index is 19 on a scale from 0–27 suggesting a substantial number of quality activities being implemented throughout the hospitals; however, also indicating future developmental potential. Minor differences observed here in the scores across pathways are the result of sampling with some hospitals not contributing data for all departments.

The index score on the involvement of patient and their patient representative in quality management overall is low. It is slightly higher for acute myocardial infarction, but the current levels of patient involvement are as expected, given this issue has only recently gained prominence in research and practice.

The score for the implementation of patient centered care strategies reflects a high level of implementation. However, given that it is based on only basic strategies to improve patient-centered care (such as implementing a policy or assessing patient views as opposed to demonstrating active engagement of patients), it also reflects further developmental potential amongst the participating hospitals. The average score is highest for deliveries and lowest for hip fracture.

The NORPEQ score is lowest for hip fracture and stroke and highest for AMI and deliveries, possibly reflecting the positive effect of treatment (or in the case of deliveries, the delivery of a healthy baby) in the latter. The perceived involvement of patients in their care process is highest for deliveries and similar for AMI, hip fracture and stroke, possibly reflecting the effect of the age-group, but also the clinical condition. This is similar for perceived health care transitions, yet here the scores for hip fracture are the lowest across the four conditions. The scores reflecting patient’s recommendation of the hospital is very high with a mean value of 3.6 on a scale from 0–4, and highest amongst patients in the AMI pathway.

Table 5 reports the results of the associational analysis between predictors and PREMs.

**Table 5:** Associations between patient-reported experience measures and predictor variables quality management systems index, patient perceived involvement in quality management and patient-centered care strategies.

	AMI		DELIVERIES		HIP FRACTURE		STROKE	
PREM / *Predictor*	b (SE)	p-value (N)	b (SE)	p-value (N)	b (SE)	p-value (N)	b (SE)	p-value (N)
**Patient experience (NORPEQ score 0–100)**								
***QMSI (Index 0–27)*^*1*^**	0.40 (0.24)	0.10 (N = 1,163)	0.11 (0.21)	0.60 (N = 1,897)	-0.40 (0.29)	0.17 (N = 1,250)	0.13 (0.26)	0.62 (N = 1,324)
***Patient Involvement in QM(Score 0–3)*^*2*^**	0.08 (1.51)	0.96 (N = 876)	-2.48 (0.86)	0.004* (N = 166)	-4.62 (1.84)	0.012* (N = 1,101)	1.02 (1.70)	0.55 (N = 1,198)
***Patient centered care strategies (Score 0–4)*^*3*^**	-1.28 (1.66)	0.44 (N = 876)	-1.19 (1.03)	0.25 (N = 1,602)	-1.06 (1.52)	0.48 (N = 1,101)	-1.46 (1.30)	0.26 (N = 1,198)
**Patient perceived involvement in their care (score 0–4)**								
***QMSI***	0.02 (0.02)	0.34 (N = 1158)	0.00 (0.01)	0.83 (N = 1902)	-0.03 (0.02)	0.08 (N = 1267)	0.0 (0.01)	0.84 (N = 1325)
***Patient Involvement in QM***	-0.03 (0.15)	0.82 (N = 870)	-0.11 (0.1)	0.033* (N = 1609)	-0.17 (0.13)	0.18 (N = 1117)	-0.06 (0.12)	0.64 (N = 1203)
***Patient centered care strategies***	-0.16 (0.16)	0.34 (N = 870)	-0.07 (0.06)	0.25 (N = 1609)	-0.08 (0.11)	0.46 (N = 1117)	-0.09 (0.10)	0.34 (N = 1203)
**Patient healthcare transition score (HCT, range 0–100)**								
***QMSI***	0.18 (0.32)	0.58 (N = 1110)	0.05 (0.23)	0.84 (N = 1823)	-0.43 (0.23)	0.06 (N = 1213)	0.19 (0.27)	0.47 (N = 1258)
***Patient Involvement in QM***	-1.16 (1.93)	0.55 (N = 832)	-1.81 (0.97)	0.63 (N = 1535)	-2.45 (1.52)	0.11 (N = 1066)	0.19 (1.79)	0.91 (N = 1153)
***Patient centered care strategies***	-1.91 (2.11)	0.37 (N = 832)	-0.97 (1.18)	0.41 (N = 1535)	-0.14 (1.27)	0.92 (N = 1066)	-0.55 (1.40)	0.69 (N = 1153)
**Patient will recommend hospital (score 0–4)**								
***QMSI***	0.02 (0.01)	0.038* (N = 1181)	0.00 (0.01)	0.50 (N = 1906)	-0.02 (0.01)	0.037* (N = 1290)	-0.00 (0.01)	0.91 (N = 1352)
***Patient Involvement in QM***	0.06 (0.06)	0.35 (N = 887)	-0.11 (0.04)	0.007* (N = 1611)	-0.15 (0.07)	0.036* (N = 1138)	0.02 (0.06)	0.75 (N = 1226)
***Patient centered care strategies***	-0.02 (0.07)	0.82 (N = 887)	0.04 (0.05)	0.44(N = 1611)	0.03 (0.06)	0.65 (N = 1138)	-0.03 (0.05)	0.61 (N = 1226)

^1^Multivariable linear mixed model, with random intercept by hospital, additionally adjusted for country, and fixed effects at the hospital level (number of beds, teaching status, and ownership) and patient level (gender, education, health literacy, and age).

^2^ Multivariable linear mixed model, with random intercept by hospital, additionally adjusted for country, and fixed effects at the hospital level (number of beds, teaching status, ownership, and QMSI), and patient level (gender, education, health literacy, and age).

^3^ Multivariable linear mixed model, with random intercept by hospital, additionally adjusted for country, and fixed effects at the hospital level (number of beds, teaching status, ownership, and QMSI), patient level (gender, education, health literacy, and age), and department level patient involvement

*significant at p<0.05 level.

Relationships between hospital and pathway level predictors (Quality Management Systems Index, Score on Patient Involvement in Quality Management and Implementation of Patient-Centered Care Strategies) and the four outcome measures (NORPEQ Patient Experience Measure, Patient Perceived Involvement in Care, Health Care Transition, and Hospital Recommendation) are presented for each of the four pathways.

Only two of the analyses reported are statistically significant at the p<0.05 level *and* have a substantive b-value. Both analyses relate to the effect of Patient Involvement in Quality Management on the NORPEQ Patient Experience measure for the deliveries (b = -2.48, p = 0.004) and hip fracture pathway (b = -4.62, p = 0.012).

Four significant associations are also observed for the relationship between predictor variable and the score on patient recommendation of the hospital; however, their corresponding b value is likely too low to be considered important.

## Discussion

This is the largest study so far to assess the complex relationship between hospital quality management systems, strategies of patient involvement and patient-centeredness in conjunction with a range of patient reported outcome measures.

Our association analysis found only a few statistically significant relationships between predictor variables and PREMs. Only two of the statistical models yielded substantive effect sizes (on the effect of patient involvement in quality management and NORPEQ score for the deliveries and hip fracture pathway); however, suggesting an inverse relationship between predictor and outcome variables. Four significant associations are also observed for the relationship between predictor variable and the score on patient recommendation of the hospital; but their corresponding b value is likely too low to be considered important. The majority of models fitted were either statistically non-significant or exhibited an effect size of no clinical or practical significance. Overall, hospital strategies and PREMs appear to be unrelated in our study.

Various plausible explanations of these unexpected results exist. First, it is possible that strategies to improve patient-centeredness are addressed in other areas of hospital administration, for example patient complaint programs, which are unconnected to the hospitals’ quality management systems and were not assessed in this study [34]. In this case our measurement strategy may be insufficient to capture all activities with a potential impact on patient-centeredness. However, it is plausible to assume that such programs typically deal only with selected groups of patients whereas our focus was on assessing whether hospital-wide governing systems exist that that aim to improve patient-centeredness for all patients.

Secondly, PREMs might simply not (or only marginally) be affected by the range of policies, procedures and strategies that we assessed and be more responsive to a direct experience of professional-patient communication. Direct personal interactions of patients with physicians or nurses are more powerful predictors of patient experience [35–36]. This observation has been also been demonstrated in the research on health care accreditation which so far failed to detect a relationship between hospitals’ accreditation status and patient satisfaction [37]. Yet, this reinforces our research question as to whether such communication processes can be supported or facilitated by hospital wide management systems. Assessment of additional constructs may be required to test this relationship (for example, are personal interactions of physicians and nurses with patients more patient-centered in organizations that promote such a patient-centered approach through their vision, policies and performance targets), but these were beyond the scope of our study.

A third explanation might be that quality management strategies and PREMs are elements of different systems (on the one hand a technocratic set of policies, principles and procedures that mainly address clinical components and resource use, and on the other an interrelated set of assumptions, expectations and expressions), which are only ‘loosely coupled’ [38]. According to Orton and Weick, a ‘loose coupling’ between a management policy and procedures in a clinical department provide the advantage of flexible organization, reaction to local (patients’) needs and local problem solving. On the other hand, in loosely coupled systems systematic changes are more difficult to implement, inhibiting an organization’s strategic development [39, 40]. More fundamentally, loose coupling may reflect a situation where hospitals created a ‘facade’ of quality management strategies to attract recognition, funding, patients and status, while not successfully pursing their implementation. In addition, management policies and procedures might be implemented in different ways and supported by different management styles. In a related study that used the same dataset, we assessed the relationships between organizational culture, organizational structure and quality management. Of the participating hospitals, 33% had a clan culture as their dominant culture type, 26% an open and developmental culture type, 16% a hierarchical culture type and 25% a rational culture type. Our findings suggest that the type of organizational culture was not associated with the development of quality management in hospitals [41].

Regarding the impact of patient involvement in quality management on PREMs the results are not so surprising. Engaging patients in quality management functions, as opposed to involving them in their own care, is a novel approach in health service delivery [42]. The seemingly counterintuitive inverse associations could be capturing a scenario where hospitals with poorer quality management were beginning to improve their patient experience. Our previous research suggests that this might be the case and calls for a more nuanced assessment of the motivation and impact of involving patients in the design and assessment of services [31, 43].

If our findings were corroborated in further research they would be of high significance for clinical practice and quality management. It is well established that higher levels of patient experience are associated with treatment adherence, better use of preventive services, health care utilization, readmissions and other outcomes [4, 8–9]. Based on this research evidence, achieving high levels of patient experience has become a cornerstone of hospital performance and has implication for the reimbursement and regulation of hospital services [44, 45].

Quality management systems have largely evolved to address clinical effectiveness and patient safety—with varying degrees of success [46–49]. Whilst it is known that hospitals employ a wide range of strategies that potentially impact patient experience, the lack of organizational infrastructure (inadequacies of data and reporting mechanisms, unclear accountabilities for monitoring, implementation and improvement, lack of clinical integration and support) may mean that not all patients benefit from such strategies and that not all strategies are subject to scrutiny, such as clinical indicator programs [50]. In order to facilitate organization-wide learning and the application of quality improvement techniques those systems that have a positive impact on PREMs should be embedded in the overall quality management system [45, 51]. Only then will a critical appraisal of possible deficiencies and achievements regarding patient-centered care be possible, similarly to hospitals’ efforts to embrace patient-safety strategies as part of their organizations’ quality management system in the last decade [52, 53].

It should be emphasized that we do not claim the findings reported here to be representative of European hospitals at large, especially considering that hospitals from Nordic Countries or Central Eastern Europe are missing or not sufficiently represented in our study. Hospitals in these countries may operate in different resource environments and exhibit different management styles while patient expectations might also differ to those included in our study. Naturally, there is variability in patient expectations both between countries as within hospitals (for example in terms of differences in expectations and experiences of patients with different acute, emergency or medical health care services, or considering the effect of patient age or socio-economic background). Our DAG guided analysis aimed to adjust for these factors (and the country effect) in order to estimate the associations between our predictor and outcome variables.

This study has a number of limitations that need to be highlighted. First, we used a cross-sectional study design which ultimately does not conclusively establish causality. We dealt with this issue by using directed acyclic graphs that guided the development of our statistical models in terms of confounding control, incorporating theory and knowledge derived from previous research findings. This approach made it possible to adjust for hospital and country characteristics in ways that allowed us to address competing explanations and plausible (non-) causal associations, while minimizing sources of bias. A second limitation is related to the sampling strategy. Although sampling was conducted randomly, a generalization to participating countries and hospitals is limited because of a possible self-selection of hospitals participating in the project. Our assessment of PREMs resonates with the literature, but the NORPEQ and HCT scores derived from our sample are slightly higher than those reported in previous research [54]. However, these higher scores should not affect the associational analysis.

## Conclusion

This is the largest studies so far to assess the complex relationship between quality management strategies and patient experience with care. Our findings suggest absence and/or wide variations in the institutionalization of quality management systems, strategies to engage patients in quality management or strategies to improve patient-centeredness of care in hospitals. Selected seemingly counterintuitive inverse associations could be capturing a scenario where hospitals with poorer quality management were beginning to improve their patient experience. Hospitals should devise organizational strategies to ensure high performance on patient experience measures similar to the achievements on clinical performance measures, whilst ensuring that these additional efforts are not to the detriment of health professionals’ interactions with patients.
